# A Comparison between the EQ-5D and the SF-6D in Patients with Chronic Obstructive Pulmonary Disease (COPD)

**DOI:** 10.1371/journal.pone.0112389

**Published:** 2014-11-07

**Authors:** Jing Chen, Carlos K. H. Wong, Sarah M. McGhee, Polly K. P. Pang, Wai-Cho Yu

**Affiliations:** 1 School of Public Health, The University of Hong Kong, Hong Kong, China; 2 Department of Family Medicine and Primary Care, The University of Hong Kong, Hong Kong, China; 3 Department of Medicine and Geriatrics, Princess Margaret Hospital, Hong Kong, China; University of Geneva, Switzerland

## Abstract

**Background:**

The appropriate use of generic preference-based measures determines the accuracy of disease assessment and further decision on healthcare policy using quality adjusted life years. The discriminative capacity of different instruments would differ across disease groups. Our study was to examine the difference in utility scores for COPD patients measured by EQ-5D and SF-6D and to assist the choice of a proper instrument in this disease group.

**Methods:**

Differences of mean utility scores of EQ-5D and SF-6D in groups defined by socio-demographic characteristics, comorbidities, health service utilisation and severity of illness were tested using Mann-Whitney test, t-test, Kruskal-Wallis test, Pearson’s correlation coefficient and ANOVA, as appropriate. The discriminative properties of the two instruments were compared against indicators of quality of life using receiver operating characteristic curves. The statistical significance of the area under the curves (AUC) was tested by ANOVA and F-statistics used to compare the efficiency with which each instrument discriminated between disease severity groups.

**Results:**

Mean utility scores of EQ-5D and SF-6D were 0.644 and 0.629 respectively in the 154 subjects included in the analysis. EQ-5D scores were significantly higher than SF-6D in groups less severe and these differences corresponded to a minimally important difference of greater than 0.03 (p<0.001). EQ-5D and SF-6D scores were strongly correlated across the whole sample (r = 0.677, p<0.001) and in pre-defined groups (r>0.5 and p<0.05 for all correlation coefficients). AUCs were above 0.5 against the indicators of health-related quality of life for both instruments. F-ratios suggested SF-6D was more efficient in discriminating cases of different disease severity than EQ-5D.

**Conclusions:**

Both EQ-5D and SF-6D appeared to be valid preference-based measures in Chinese COPD patients. SF-6D was more efficient in detecting differences among subgroups with differing health status. EQ-5D and SF-6D measured different things and might not be used interchangeably in COPD patients.

## Background

There is an increasing need to express effectiveness of interventions in terms of quality-adjusted life years (QALYs) in health economic evaluations. The QALY accounts for both length of life and health-related quality of life (HRQoL) in a single outcome measure. Preference-based measures of health are designed to construct QALYs. They consist of two parts, one a standardized descriptive system for people to describe their own health state, the other an algorithm for assigning values to each state. The algorithm is usually based on valuations from a general population sample of adults which are derived using techniques such as standard gamble (SG) or time trade off (TTO). Preference-based measures score health states on a scale of 0 to 1 equivalent to death and full health respectively [1].

There are several preference-based measures including the EQ-5D [2], the 15D [3], Health utility index (HUI) [4], Quality of Well-Being (QWB) [5] and most recent of all, the SF-6D [6]. The EQ-5D is currently the most popular preference-based instrument, used in about half of published studies [7]. It is a two part instrument, with the first part describing problems in five dimensions with three response levels for each. The second part consists of a vertical visual analogue scale (VAS) with zero at the bottom referring to the worst health state and 100 at the top referring to the best health state. The participant pointed to the level on the scale indicating their overall health on the day of the interview. The five dimensions (mobility, self-care, usual activities, pain/discomfort, anxiety/depression) each with three levels (no problems, some problems, extreme problems) can thus define 243 (3^5^) different health states [1]. Using the TTO technique, country specific scoring algorithms have been developed for the EQ-5D in UK [8], Norway [9], and Japan [10]. The EQ-5D has been translated into Chinese and used in China, Taiwan, Hong Kong, Singapore and Malaysia [11]. The SF-6D, which is increasingly used, was developed from the SF-36 [6]. Six dimensions (physical functioning, role limitation, social functioning, pain, mental health, vitality) were retained from the original SF-36, each with 4 to 6 levels and it can define 18 000 (6*6*5*5*5*4) different health states. Using the standard gamble (SG) technique, a scoring algorithm for the SF-6D was developed for the UK [6], [12].

The EQ-5D and the SF-6D differ from one another in terms of their valuation techniques, classification systems, dimensions and items covered. There is therefore a possibility that they will generate a different utility score for a specific health state. Several studies have directly compared these two instruments in different populations, and these generally show disparities in the utility scores derived from the EQ-5D and the SF-6D [12]–[20]. The EQ-5D tends to generate higher scores than the SF-6D in healthy population or patients with mild conditions and vice versa in less healthy populations or among patients with severe conditions [13], [17], [19], leading to variation in the estimation of utility scores and QALYs which could influence decisions taken on resource use [21]–[23].

Harper *et al* showed that the SF-36 performed better in detecting changes in HRQoL in patients with mild COPD in outpatient settings than did the EQ-5D [24]. However, no study to our knowledge has directly compared the performance of EQ-5D and SF-6D for those with more severe stages of COPD. In order to assist clinicians and researchers in choosing the most appropriate instrument for discriminative and evaluative purposes we performed a head-to-head comparison of the EQ-5D and the SF-6D in a group of severe and very severe COPD patients. COPD was chosen because it is known to have a significant adverse impact on HRQoL [25]–[30]. The burden of the disease in terms of morbidity and mortality is projected to increase in the next few decades in East Asia and elsewhere making the ability to determine QALY loss important [31], [32], thus a precise measure is required in estimate of QALYs and to better inform the healthcare policy using quality-adjusted life years as outcome.

## Methods

### Sample and data collection

The study was conducted between September 2010 and May 2011 in a respiratory specialist out-patient clinic (SOPC) in a public hospital (Princess Margret Hospital) in Hong Kong. All the patients with COPD who regularly attended the SOPC were screened by their medical records for the study. Patients who had severe (post-bronchodilator FEV_1_ 30–49% of predicted) or very severe (post-bronchodilator FEV_1_% < 30% of predicted) COPD according to the Global Obstructive Lung Disease (GOLD) standard [33] during the past one year were included. Those who had significant comorbidities which prevented their participation, such as dementia or Parkinson's disease, who did not have a pulmonary function test result within the past one year or who did not speak Cantonese were excluded and all the eligible patients were invited to participate in the study. From 166 patients approached, 2 patients were incommunicable (one was deaf and one was unable to speak), 5 refused to participate, and 159 (95.8%) provided written consent to participate and were interviewed by trained interviewers. Five of the interviewed participants were excluded from the analysis because their post FEV_1_ was 50% of predicted which classified their severity stage as moderate. Thus, 154 patients were included in the final data analysis.

Subjects completed the respiratory disease specific Saint George’s Respiratory Questionnaire (SGRQ), the SF-6D and the EQ-5D. They also answered questions on socio-demographic information (sex, age, education, housing type and income), self-rated health status (“Compared with past 12 months, what do you think about your present health condition? Better, The same, Worse”) and life style information (e.g. smoking). The SF-6D and the EQ-5D were asked in a random sequence to avoid possible order effects. Utilization of health services (hospital admissions in the past 12 month) and presence of comorbidities (i.e. other than COPD, whether being diagnosed with hypertension, heart diseases, pneumonia, diabetes, asthma, rheumatoid arthritis, liver disease, cancers and other chronic diseases) were extracted directly from patients’ medical records by one research nurse in the SOPC.

The study was approved by the Kowloon West Cluster Clinical Research Ethics Committee, Hong Kong. All participants provided written informed consent to participate in this study.

### HRQoL Measures

The Cantonese Chinese version of the EQ-5D and the Chinese (Hong Kong) SF-6D questionnaire were used to measure the Health Related Quality of Life (HRQoL) of the sample patients [34]. Because a Hong Kong specific scoring algorithm was not available for the EQ-5D, the UK one was used for which direct valuations of 43 health states had been elicited from a sample of the general population using TTO and the theoretic utility scores ranged from −0.59 to 1 [35].

Direct valuations for 196 health states from the SF-6D HK had been valued by a representative sample of the Hong Kong population (n = 582) using SG and the theoretic utility scores ranged from 0.315 to 1 [36]. This algorithm was employed in the present study to generate utility scores but, in order to compare with scores from the EQ-5D, the UK scoring algorithm for the SF-6D [6] was also used.

The Hong Kong Chinese version of the St. George’s Respiratory Questionnaire (SGRQ) was included as a measure of disease specific quality of life. The SGRQ contains 76 weighted responses divided into three sections covering distress due to respiratory symptoms, disturbance of physical activity and overall impact on daily life and well-being. A total score from 0 to 100 is summed from weighted positive responses and a higher score indicates worse HRQoL [37]. The Chinese version of the SGRQ had already been validated in patients with COPD in Hong Kong [38] and, although the weights applied to the responses have not been developed in Hong Kong, they appear to be similar in different countries and in different languages [39] so cultural or linguistic factors may not greatly affect responses to this questionnaire [40].

### Statistical analysis

Utility scores for the EQ-5D and the SF-6D were both estimated using UK-derived scoring algorithms for direct comparison and a SGRQ total summary score was calculated. Possible ceiling and floor effects, represented by the proportion of participants with the best and worst theoretical scores respectively, were identified. Correlation between the SGRQ scores and EQ-5D or SF-6D or EQ-VAS scores were tested using Pearson’s correlation coefficient.

The EQ-5D, SF-6D and SGRQ mean scores were compared for different groups defined by levels of socio-demographic variables, comorbidities or health service utilisation. Where the relevant variable had two levels, this between-group comparison used the Mann-Whitney test for the EQ-5D or t-test for the SF-6D and SGRQ. Where the relevant variable had three or more levels, this comparison used the Kruskal-Wallis test for the EQ-5D scores or the one way analysis of variance (ANOVA) for SF-6D scores and SGRQ. Association between the EQ-5D and SF-6D mean scores of each level of each variable was identified using Pearson’s correlation coefficient and statistical significance of the difference was assessed by paired-sample t-test. A minimally important difference (MID) was defined as a difference of at least 0.03 between the EQ-5D and the SF-6D mean scores [20], [41], [42].

To determine whether the mean utility scores of the EQ-5D and SF-6D followed the pattern that less severe patients had higher scores on the EQ-5D and more severe patients scored higher on SF-6D, the correlations between EQ-5D and SGRQ as well as SF-6D and SGRQ were tested by simple linear regression. The difference between the SF-6D and EQ-5D scores was regressed to SGRQ scores to further explore whether the two utility scores vary with severity differently. SGRQ was used as an external indicator of disease severity in COPD patients.

The discriminative properties of the EQ-5D and SF-6D were compared using receiver operating characteristic (ROC) curves. The area under the ROC curve (AUC) indicates the possibility of correctly discriminating between dichotomized groups (e.g. “severe” vs. “very severe”). In our analysis, the performance of the EQ-5D and SF-6D was evaluated against a couple of commonly used external indicators of HRQoL: the EQ-VAS scores and the SGRQ summary scores. These indicators were dichotomized using different cut-off points. EQ-VAS scores and SGRQ scores was grouped according to the percentiles of the score distribution. The statistical significance of the AUC (F-statistic) was tested by ANOVA for each instrument. F-statistics for the SF-6D and EQ-5D were compared by dividing the F-statistic of the SF-6D by that of the EQ-5D (F-ratio). A value greater than one indicates that the SF-6D is more efficient than the EQ-5D at detecting differences in quality of life. The significance level was set as p<0.05 and all analyses were performed using STATA (StataCorp. 2013. Stata Statistical Software: Release 13. College Station, TX: StataCorp LP).

## Results

Almost all the patients were male (98.7%), 65.0% were in the severe COPD stage and, on average, had been diagnosed with COPD for 7 years (SD 4.4) (Table 1). More than 80% were ex-smokers while 12% were current smokers. Fewer than 30% had secondary education or higher and 68.8% had no family income while 67.5% of them were with comorbid conditions and 72.1% had been admitted to hospital at least once in the past 12 months. Around three-quarters of the group (74.0%) reported their health to be worse compared with the previous year.

**Table 1:** Characteristics of the study sample (n = 154).

	Severe COPD		Very severe COPD		Total	
	n	%	n	%	n	%
**Number of patients**	100	65.0	54	35.0	154	100.0
**Age (yrs, mean ±, sd)** ¶	74.8±7.9		69.4±7.4		72.9±8.1	
**Gender (male)**	98	98	54	100	152	98.7
**COPD duration**						
(yrs, mean ± sd)	6.8±4.2		7.9±4.8		7.2±4.4	
**Lung function: post FEV_1_% (mean ± sd)** ¶	38.1±5.7		22.6±5.1		32.7±9.2	
**Education**						
No/pre-primary	30	30.0	7	13.0	37	24.0
Primary	49	49.0	31	57.4	80	52.0
Secondary	20	20.0	15	27.8	35	22.7
Post-secondary	1	1.0	1	1.9	2	1.3
**Monthly family income (HK$)**						
No income	73	73.0	33	61.1	106	68.8
<$10,000	26	26.0	20	37.0	46	29.9
>$10,000	1	1.0	1	1.9	2	1.3
**Housing**						
Public or aided	76	76.0	47	87.0	123	79.9
Private or Old people’s home	24	24.0	7	13.0	31	20.1
**Smoking** ‡						
Current smoker	16	16.0	3	5.6	19	12.3
Ex-smoker	83	83.0	47	87.0	130	84.4
Never smoker	1	1.0	4	7.4	5	3.3
**Comorbidity** ∏						
No	35	35.0	15	27.8	50	32.5
Yes	65	65.0	39	72.2	104	67.5
**Hospital admission in the past 12 month**						
No	33	33.0	10	18.5	43	27.9
Yes	67	67.0	44	81.5	111	72.1
**Perceived health (compared to previous year)**						
Better	11	11.0	4	7.4	15	9.7
Same	18	18.0	7	13.0	25	16.2
Worse	71	71.0	43	79.6	114	74.0

∏ Presence with any following disease: hypertension, heart diseases, pneumonia, diabetes, asthma, rheumatoid arthritis, liver disease, cancers and other chronic diseases.

¶ p<0.001, significance was tested by t-test.

‡ p<0.05, significance was tested by chi-square test.

Note: COPD  =  Chronic Obstructive Pulmonary Disease.

**Figure 1:**
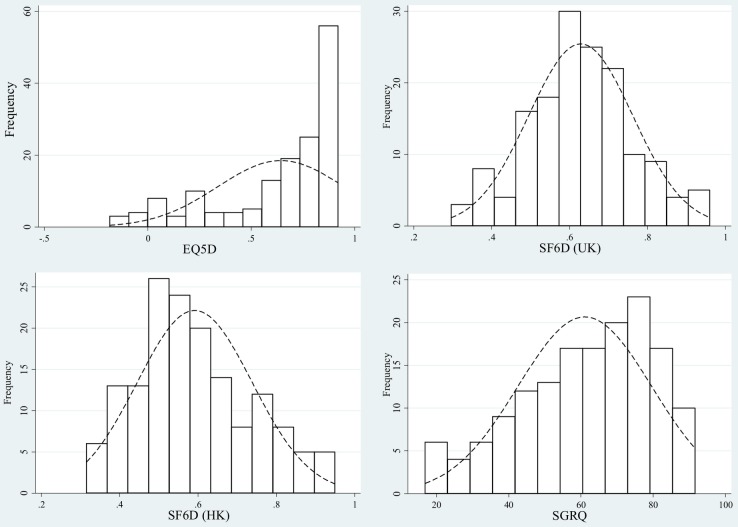
Distribution of EQ-5D, SF-6D (UK), SF-6D (HK) and SGRQ scores.

The mean scores on the EQ-5D and the SF-6D (UK) were 0.644 and 0.629 respectively. The distribution of EQ-5D, SF-6D (UK and HK) and SGRQ scores were generally skewed, particularly noticeable for the EQ-5D (Figure 1). The EQ-5D showed a ceiling effect with 22.1% of patients reporting the highest possible scores. No ceiling effect was observed in the SF-6D or SGRQ. Both EQ-5D and SF-6D scores were negatively associated with SGRQ since high scores indicated good HRQoL on EQ-5D and SF-6D but poor HRQoL on the SGRQ. Correlation was higher between SF-6D (HK and UK) and SGRQ (r = −0.745 and −0.728, p<0.001) than between EQ-5D and SGRQ (r = −0.583, p<0.001) (Table 2).

**Table 2:** Distribution of EQ-5D, SF-6D (UK & HK), EQ-VAS and SGRQ scores in the sample subjects and their correlations with SGRQ.

Measures	Theoretical range	Observed range	Mean ± sd	Ceiling effect (%)	Floor effect (%)	Pearson correlation¶
**EQ-5D**	−0.590, 0.919	−0.184, 0.919	0.644±0.306	34 (22.1)	0	−0.583
**SF-6D (HK)**	0.315, 1	0.315, 0.950	0.591±0.147	0	2 (1.3)	−0.745
**SF-6D (UK)**	0.296, 1	0.296, 0.960	0.629±0.133	0	1 (0.6)	−0.728
**EQ-VAS**	0, 100	0, 100	55.28±20.42	4 (2.6)	5 (3.2)	−0.437
**SGRQ**	100, 0	91.62, 16.84	61.07±18.53	0	0	–

¶ EQ-5D, SF-6D and EQ-VAS correlated with SGRQ; p<0.001 for all Pearson’s correlation coefficient.

Note: SGRQ  =  Saint George’s Respiratory Questionnaire.

**Figure 2:**
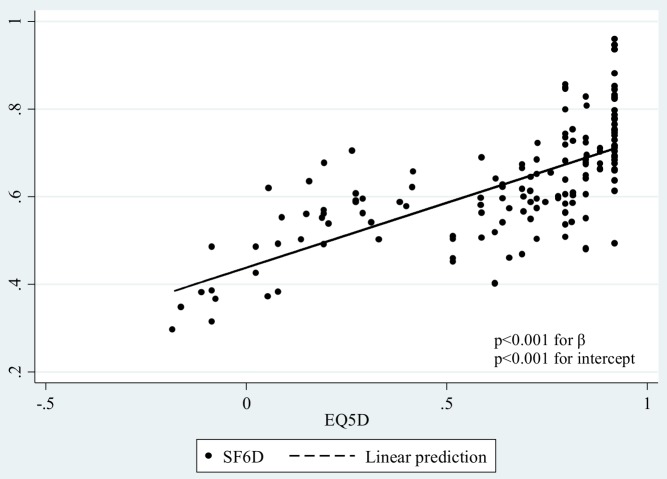
Scatter plot of EQ-5D and SF-6D.

The EQ-5D and SF-6D mean scores were strongly correlated across the whole sample (r = 0.677, p<0.001) and a scatter plot further showed this when regressing EQ-5D to SF-6D (the coefficient ß = 0.30, p<0.001, and the constant  =  0.44, p<0.001) (Figure 2). In subgroups defined by disease severity, socio-demographic characteristics and utilization of health services, the EQ-5D and SF-6D mean scores were strongly correlated as well (Table 3). Both measures were sensitive to differences within subgroups in disease severity, hospital admission and presence or not of comorbidities and differences detected were statistically significant.

**Table 3:** EQ-5D and SF-6D (UK) mean scores, mean score difference between EQ-5D and SF-6D, and correlation between EQ-5D and SF-6D scores by group.

		Mean scores			Mean score difference (EQ-5D – SF-6D)		Pearson correlation between EQ-5D and SF-6D	
Variables	n (%)	SGRQ	EQ-5D	SF-6D	Estimate	p value for md#	Estimate	p value for pcc
**Total sample**	154	61.07	0.644	0.629	0.015	0.21	0.677	<0.001
**Stage of COPD**								
Severe	100 (64.9)	57.76	0.686	0.646	0.041	0.03	0.688	<0.001
Very severe	54 (35.1)	67.19	0.565	0.597	−0.032	0.19	0.633	<0.001
*p* value§		0.001	0.007	0.015				
**Age**								
50–59	12 (7.8)	58.27	0.808	0.650	0.158	<0.001	0.684	0.014
60–69	39 (25.3)	61.69	0.655	0.611	0.044	0.12	0.652	<0.001
70–79	75 (48.7)	61.43	0.611	0.629	−0.018	0.27	0.716	<0.001
80+	28 (18.2)	60.43	0.646	0.643	0.002	0.48	0.701	<0.001
* p* value∏		0.98	0.54	0.72				
**Education**								
No/pre-primary	37 (24.0)	62.32	0.618	0.610	0.008	0.42	0.784	<0.001
Primary	80 (52.0)	62.72	0.657	0.634	0.022	0.19	0.657	<0.001
Secondary	35 (22.7)	56.76	0.630	0.630	0.0003	0.50	0.604	<0.001
Post-sec/degree	2 (1.3)	47.20	0.833	0.708	0.125	0.08	1.0000	<0.001
* p* value∏		0.28	0.82	0.67				
**Hospital admission**								
No	43 (27.9)	52.07	0.772	0.688	0.084	<0.001	0.533	<0.001
Yes	111 (72.1)	64.55	0.594	0.605	−0.011	0.32	0.689	<0.001
* p* value§		<0.001	0.002	<0.001				
**Comorbidities**								
No	50 (32.5)	59.77	0.706	0.650	0.056	0.03	0.745	<0.001
Yes	104 (67.5)	61.69	0.614	0.618	−0.004	0.43	0.642	<0.001
*p* value§		0.27	0.04	0.04				

# Significance test by paired-samples t-test.

§ P value for between-group comparison, significance test by t-test for SF-6D and SGRQ; Mann-Whitney test for EQ-5D.

∏ P value for between-group comparison, significance test by ANOVA for SF-6D and SGRQ; Kruskal-Wallis test for EQ-5D.

Note: COPD  =  Chronic Obstructive Pulmonary Disease; SGRQ  =  Saint George’s Respiratory Questionnaire.

**Figure 3:**
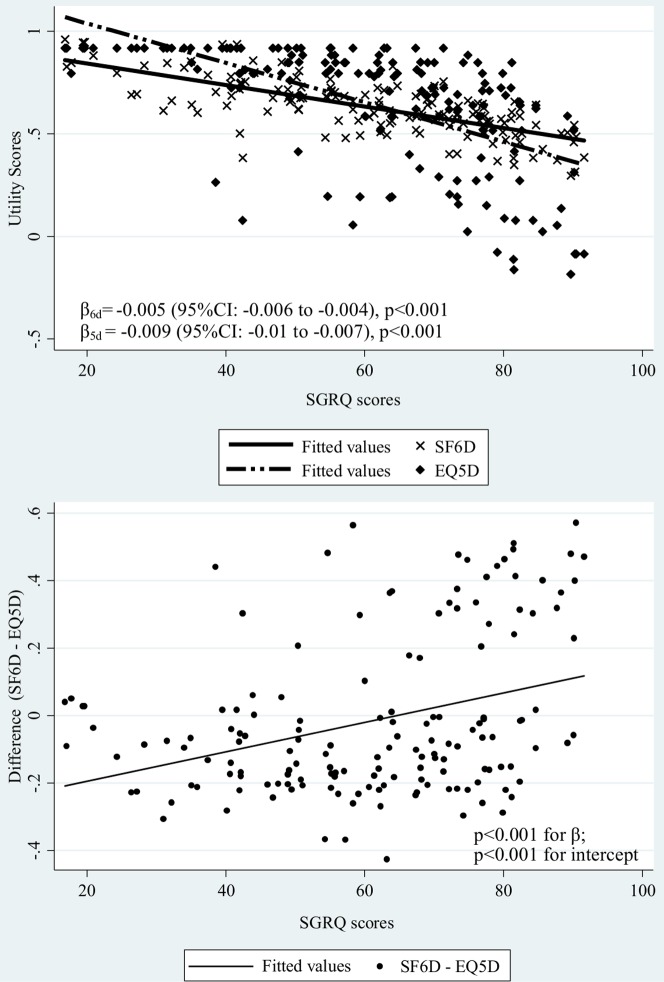
Scatter plots of SF-6D vs. SGRQ, EQ-5D vs. SGRQ (above) and difference (SF-6D – EQ-5D) vs. SGRQ (below).

Direct comparison of the EQ-5D and SF-6D showed that in the less severe cases indicated by lower SGRQ scores, the EQ-5D scores were higher than SF-6D (Figure 3 above). On the contrary, the SF-6D scores were higher than EQ-5D in more severe cases indicated by a higher SGRQ scores. There appeared a cross-over of these two utility scores. The correlation between the difference of the SF-6D and EQ-5D (SF-6D – EQ-5D) and SGRQ score was positive (β = 0.004, p<0.001) (Figure 3 below). The difference in most cases (88.3%) were greater than the MID of 0.03.

The AUC showed the discriminative ability of the EQ-5D and SF-6D to distinguish between very severe and severe COPD as defined by EQ-VAS and SGRQ summary scores. Against both indicators of HRQoL, the AUC scores for both instruments were above 0.5 suggesting that they can detect the difference between severe and very severe cases (Table 4). For most of the comparisons, the SF-6D generated a larger AUC than the EQ-5D. Most of the F-ratios was greater than 1 which showed SF-6D to be more efficient in discriminating cases of differed disease severity than EQ-5D.

**Table 4:** Area under receiver operating characteristic curves (AUC) for EQ-5D and SF-6D with different cut-off points in external indicators.

	SF-6D				EQ-5D			
	AUC	95%CI		F statistics	AUC	95%CI		F statistics	F ratio
**EQ-VAS scores**									
> = 50 vs. <50	0.718	0.631	0.804	18.37	0.724	0.629	0.818	24.13	0.76
> = 60 vs. <60	0.672	0.587	0.756	16.21	0.649	0.562	0.735	14.11	1.15
> = 70 vs. <70	0.695	0.602	0.788	18.02	0.652	0.559	0.746	8.15	2.21
> = 80 vs. <80	0.733	0.610	0.856	13.11	0.687	0.563	0.812	5.32	2.46
> = 90 vs. <90	0.763	0.592	0.934	6.41	0.755	0.610	0.899	4.13	1.55
**SGRQ scores**									
>49 vs. < = 49	0.864	0.789	0.938	67.26	0.826	0.749	0.902	23.88	2.82
>64 vs. < = 64	0.835	0.769	0.900	66.30	0.850	0.788	0.911	54.97	1.21
>77 vs. < = 77	0.867	0.809	0.925	60.02	0.846	0.784	0.908	54.34	1.10

Note: AUC  =  Area under receiver operating characteristics curves; CI  =  Confidence interval; SGRQ  =  Saint George’s Respiratory Questionnaire.

## Discussion

It is important to compare the available preference-based measures of health because the choice of instrument can greatly influence the eventual decision-making based on the results. Different instruments can have differing sensitivity to disease-related quality of life impacts and can display ceiling or floor effects in some populations. This comparative study of the EQ-5D and the SF-6D in people with severe or very severe COPD confirmed the ceiling effect of the EQ-5D, that is, a substantial group of subjects scored the highest possible score, even though all had COPD classed as severe by lung function tests. This ceiling effect of the EQ-5D has been noted in other studies [12], [13], [16]. Both of the instruments demonstrated a moderate capacity to distinguish between previously defined groups in terms of disease severity as assessed by lung function, by whether they had a recent hospital admission and by whether they had any comorbidities. Neither instrument was found to be clearly superior. When compared with the SGRQ, the SF-6D showed stronger correlation coefficients and generated a larger AUC than EQ-5D implying that SF-6D may be better at reflecting the health status of those with severe COPD.

Both the EQ-5D and the SF-6D discriminated well between pre-defined subgroups with mainly significant differences between mean scores for groups defined by disease severity, hospital admissions and presence of comorbidities. Thus there is evidence of construct validity for each measure in identifying HRQoL of people with severe and very severe COPD. The EQ-5D and SF-6D were strongly positively correlated (Pearson’s correlation coefficient >0.5 and statistically significant) throughout the entire sample and pre-defined subgroups. Despite this, the difference in mean scores on the two measures in pre-defined subgroups often constituted a MID.

Even though the EQ-5D and SF-6D generated similar mean scores in our sample, the distributions were very different. The SF-6D utility scores followed a relative normal distribution, unlike the EQ-5D scores which were negatively skewed with a clustering at the maximum score of one. It was clear from the analysis that the EQ-5D appeared with larger ceiling effect (22.1%) compared with the SF-6D (UK and HK) in our sample. Previous studies in populations with chronic diseases have also reported ceiling effects for the EQ-5D [43]–[45]. It suggested that the EQ-5D, in which 22.1% of our sample patients scored the highest possible score, may not be as efficient as SF-6D in reflecting the disease severity. Further direct comparison of the EQ-5D and the SF-6D scores against disease severity indicated by SGRQ scores also confirmed this. The relationship observed between the SF-6D and SGRQ differed from that between the EQ-5D and SGRQ, where the SF-6D scored higher in more severe cases and the EQ-5D scored higher in less severe cases. This may be due to valuation techniques, which is TTO for the EQ-5D, as well as scaling properties. Others have found that SG generally produces higher values than TTO for the same condition [35], [46] but TTO usually gives higher values than SG for milder health states [47]. This suggests that there might be a “cross-over” point for TTO and SG scores along the axis of illness severity and hence, also, for EQ-5D and SF-6D values. We did indeed see this crossover point in our study.

Moreover, consistent with other studies [19], [21], [45], the SF-6D was more efficient in detecting HRQoL differences between subgroups than the EQ-5D with the SF-6D generating larger AUCs and the F-ratio of SF-6D to EQ-5D being greater than 1 in most cases in our study. Specifically, Harper *et al* had already shown that SF-36 was better than EQ5D in detecting minor changes in HRQoL for those with mild COPD in outpatient settings [24] and our study adds evidence that in severe and very severe stages of COPD, the EQ-5D and the SF-6D show different discriminative capacities with SF-6D being more efficient in detecting changes in HRQoL. However, some studies have still preferred to use the EQ-5D for chronic disease in clinic settings and for comparing HRQoL pre and post-intervention [43]. Stel *et al* found that the EQ-5D had a substantially lower percentage of missing data than the SF-6D both at baseline and post-intervention assessments when assessing the impact of different surgical interventions on patients with coronary heart disease. Our study did not find such a difference in completeness. Thus along with Harper *et al* [24], the SF-6D would be a more precise measurement in estimate of QALYs in patients with COPD than the EQ-5D. It is evident that the EQ-5D and the SF-6D yield different utility values and measured different things in COPD patients. There two instruments might not be used interchangeably, so a careful choice needs to be made. It is therefore important for clinical and health technology assessment authorities to choose the most appropriate measurement to better inform the healthcare policy using QALYs as outcome. More comparative studies would be useful.

### Strengths and limitations

To our knowledge, our study was the first head to head comparison of the SF-6D and the EQ-5D among Chinese subjects with a severe chronic disease. Classifying health status using SGRQ and EQ-VAS scores could capture the underlying disease severity and provide reliable and validated external measures of HRQoL. Existing clinical conditions and related health care utilization data were extracted directly from medical records which minimized errors and bias in recall.

However, there were several potential limitations in our study. Firstly, the UK-derived scoring algorithms were used for comparing the EQ-5D and the SF-6D since there was no local algorithm available for the EQ-5D. This may fail to reflect the true preference in a Chinese population. Secondly, our sample was mainly male (98%) which may limit the external validity of our results. However, evidence on any gender difference in HRQoL is not conclusive with many studies showed non-significant differences [25], [27], [29], [48], [49]. A local study did not find any difference in the HRQoL of males and females with COPD who were attending community health centers (unpublished data). Finally, the cross-sectional nature of this study renders it unable to test the ability of the instruments to detect changes in HRQoL.

## Conclusions

Both the EQ-5D and the SF-6D appeared to be valid and discriminative preference-based measures of health in Chinese patients with severe and very severe COPD. Our study added evidence that the SF-6D was more efficient in detecting differences among subgroups with differing disease severity of COPD. The EQ-5D and the SF-6D measured different things and might not be used interchangeably in COPD patients. Further research would be needed to determine other psychometric properties of the EQ-5D and the SF-6D such as responsiveness to assess ability to detect longitudinal changes.
